# Co-Doping Effects on the Electronic and Optical Properties of β-Ga_2_O_3_: A First-Principles Investigation

**DOI:** 10.3390/ma18092005

**Published:** 2025-04-28

**Authors:** Ya-Rui Wang, Su-Zhen Luan

**Affiliations:** School of Communication and Information Engineering, Xi’an University of Science and Technology, Lintong Campus, Xi’an 710699, China; 15991618639@163.com

**Keywords:** β-Ga_2_O_3_, band structure, optical absorption, co-doping

## Abstract

To meet the demands for functional layers in inverted flexible perovskite solar cells, high-performance formamidinium-based perovskite solar cells, and high-performance photodetectors in future applications, it is crucial to appropriately reduce the bandgap of third-generation wide-bandgap semiconductor materials. In this study, we first optimized doping sites through Ag-Cl and Ag-S configurations to establish stable substitution patterns, followed by density functional theory (DFT) calculations using the Generalized Gradient Approximation with the Perdew–Burke–Ernzerhof (GGA-PBE) functional, implemented in the Vienna Ab initio Simulation Package (VASP). A plane-wave basis set with a cutoff energy of 450 eV and a 3 × 4 × 3 Γ-centered k-mesh were adopted to investigate the effects of Mg-Cl, Mg-S, Zn-Cl, and Zn-S co-doping on the structural stability, electronic properties, and optical characteristics of β-Ga_2_O_3_. Based on structural symmetry, six doping sites were considered, with Ag-S/Cl systems revealing preferential occupation at octahedral Ga(1) sites through site formation energy analysis. The results demonstrate that Mg-Cl, Mg-S, Zn-Cl, and Zn-S co-doped systems exhibit thermodynamic stability. The bandgap of pristine β-Ga_2_O_3_ was calculated to be 2.08 eV. Notably, Zn-Cl co-doping achieves the lowest bandgap reduction to 1.81 eV. Importantly, all co-doping configurations, including Mg-Cl, Mg-S, Zn-Cl, and Zn-S, effectively reduce the bandgap of β-Ga_2_O_3_. Furthermore, the co-doped systems show enhanced visible light absorption (30% increase at 500 nm) and improved optical storage performance compared to the pristine material.

## 1. Introduction

In recent years, the large-scale development of wide-bandgap semiconductor materials has opened up new possibilities for the advancement of next-generation power electronic devices and radio-frequency equipment [1,2,3,4,5]. Among various wide-bandgap semiconductors, Ga_2_O_3_ has emerged as a prominent third-generation semiconductor material due to its excellent bandgap characteristics and high breakdown field strength, making it a focal point of research [6,7]. The experimental realization of β-Ga_2_O_3_’s superior properties relies heavily on advanced synthesis techniques, with metalorganic chemical vapor deposition (MOCVD) being the most widely adopted method for producing high-quality epitaxial layers. Studies have shown that Ga_2_O_3_ exists in five crystalline phases: α, β, δ, γ, and ε [8,9]. Among these, β-Ga_2_O_3_ (monoclinic structure) stands out due to its exceptional stability [10]. This material not only exhibits superior breakdown field strength and electron mobility but also demonstrates excellent ultraviolet transparency, remarkable photocatalytic activity, and outstanding physicochemical stability. Additionally, its relatively low fabrication cost makes it highly valuable for a wide range of applications [11,12]. As a promising candidate for next-generation semiconductors, gallium oxide (Ga_2_O_3_) has garnered significant attention in various fields due to its outstanding physical properties. These include applications in Schottky diodes [13,14], solar-blind ultraviolet photodetectors [15,16,17], photocatalysis [18], and high-power transistors [19,20,21].

In recent years, researchers have focused on enhancing the electronic and optical properties of β-Ga_2_O_3_ [22]. Among various modification methods, alloy doping has been proven to be an effective strategy for tuning the performance of semiconductor materials [23,24]. Yan et al. [25] demonstrated that co-doping with electron-deficient metals and nitrogen not only reduces the acceptor levels in β-Ga_2_O_3_ but also provides a feasible approach for achieving p-type β-Ga_2_O_3_. Additionally, Srivastava et al. [26] systematically investigated the effects of indium and nitrogen single doping on the electronic structure and optical properties of β-Ga_2_O_3_ using first-principles calculations. Their results revealed that increasing the concentration of indium dopants leads to a significant widening of the material’s bandgap. Hong and Zheng et al. [27] experimentally confirmed that constructing a Ni_x_O/SiN_x_/Ga_2_O_3_ multilayer heterostructure in β-Ga_2_O_3_ devices significantly improves the forward conduction characteristics of diodes while effectively reducing the device’s on-resistance. Fang et al. [28] developed Ga_2_O_3_: Cr^3^⁺ phosphors with a quantum yield of up to 92.4% via a Cr^3^⁺ single doping strategy, and their unique double spin-forbidden jump (R1/R2) emission properties were successfully applied to plant photosensitization modulation. The material was experimentally confirmed to significantly promote plant growth in LED-driven agricultural and horticultural scenes, revealing the value of Ga_2_O_3_-based materials for interdisciplinary applications in biophotonics. In the field of doping modification, Ma and Lin’s team explored the effects of Al-N and In-N co-doping on β-Ga_2_O_3_ [29]. Their findings indicated that the Al-N co-doped system exhibits lower defect formation energy and shallower transition levels compared to single N doping. Notably, the In-N co-doped system demonstrated superior performance, with further reduced transition layer depth and significantly increased carrier concentration. Based on the periodic trends, we note that sulfur (S) belongs to the same group (VIA) as oxygen, while chlorine (Cl), as a group VIIA element, has a valence electron structure similar to oxygen. In addition, the elements have similar atomic radii, which ensures the stability of the structure and is not prone to lattice distortion. These characteristics make them promising candidates for modulating the electronic band structure and optoelectronic properties of β-Ga_2_O_3_. Although magnesium (Mg) and zinc (Zn) have been confirmed as effective dopants, the mechanisms underlying the effects of Mg-Cl, Mg-S, Zn-Cl, and Zn-S co-doping on the properties of β-Ga_2_O_3_ remain insufficiently studied. Therefore, this research focuses on investigating the synergistic effects of Mg and Zn co-doped with Cl and S on the electronic structure and optical properties of β-Ga_2_O_3_.

In this study, we employed first-principles calculations based on density functional theory to systematically investigate the modulation mechanisms of Mg-Cl, Mg-S, Zn-Cl, and Zn-S co-doping on the stability, electronic properties, and optical performance of β-Ga_2_O_3_. By constructing nine computational models with different doping sites and structural symmetries, we thoroughly analyzed the physicochemical properties of the co-doped systems. The results reveal that all co-doped configurations exhibit excellent thermodynamic stability. Notably, all four co-doping systems effectively modulate the band structure of β-Ga_2_O_3_, significantly reducing its bandgap. Further analysis demonstrates that these co-doped systems not only enhance the electrical conductivity of the material but also improve its absorption efficiency in the visible light range.

## 2. Theoretical Method

### 2.1. Structural Model

**Figure 1:**
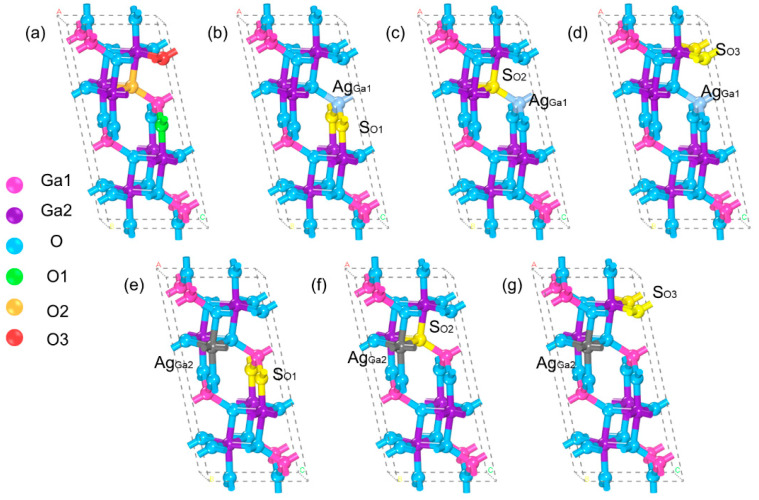
(**a**) Schematic diagram of the β-Ga_2_O_3_ crystal structure. (**b**–**g**) Schematic illustrations of the doping sites. Figures (**a**,**b**,**c**), where light blue atoms indicate metal Ag atoms doped with Ga1 sites, and grey atoms in figures (**e**,**f**,**g**) indicate metal Ag atoms doped with Ga2 sites.

The β-Ga_2_O_3_ crystal exhibits a monoclinic structure with a space group of C2/m [30]. According to previous studies, its lattice parameters are a = 12.23 Å, b = 3.04 Å, and c = 5.80 Å, with a c/a ratio of 0.474 [31]. From the perspective of crystal structure, β-Ga_2_O_3_ contains two distinct Ga atomic sites and three O atomic sites, labeled as Ga1, Ga2, O1, O2, and O3, respectively. During the doping process, metal elements tend to substitute Ga sites, while non-metal elements primarily occupy O sites. This substitution behavior is closely related to the valence electron density of the dopants. Notably, in co-doped systems, both metal and non-metal elements simultaneously replace Ga and O sites. To illustrate this structural feature, Figure 1a–g systematically present the crystal structure models of intrinsic β-Ga_2_O_3_ and its co-doped systems, highlighting the possible occupation sites of different dopants.

### 2.2. Calculated Method

To identify the optimal doping sites, the study first calculated the system energy based on a 1 × 1 × 1 unit cell model. For the (metal, non-metal) co-doped β-Ga_2_O_3_ systems, we employed first-principles calculations and constructed a 1 × 3 × 2 supercell model to systematically investigate their total energy, electronic structure characteristics, and optical properties. The calculations were performed using the Vienna Ab initio Simulation Package (VASP 6.3.2) [32,33]. In terms of computational parameters, the Perdew–Burke–Ernzerhof (PBE) method within the generalized gradient approximation (GGA) framework was used to optimize the structures of all doped systems [34,35]. The ion–electron interactions were described using ultrasoft pseudopotentials [36]. The valence electron configurations of the elements were set as follows: Ga (3d^10^4s^2^4p^1^), O (2s^2^2p^4^), Mg (3s^2^), and Zn (4s^2^). The plane-wave cutoff energy was set to 450 eV, and a 3 × 4 × 3 k-point mesh was adopted. The self-consistent field (SCF) convergence criterion was strictly controlled to within 1 × 10^−8^ eV/atom. During the geometric optimization process, the lattice constants and atomic coordinates of all co-doped systems were fully relaxed [37,38].

## 3. Results and Discussion

### 3.1. Crystal Structure and Formation Energy Analysis

**Figure 2:**
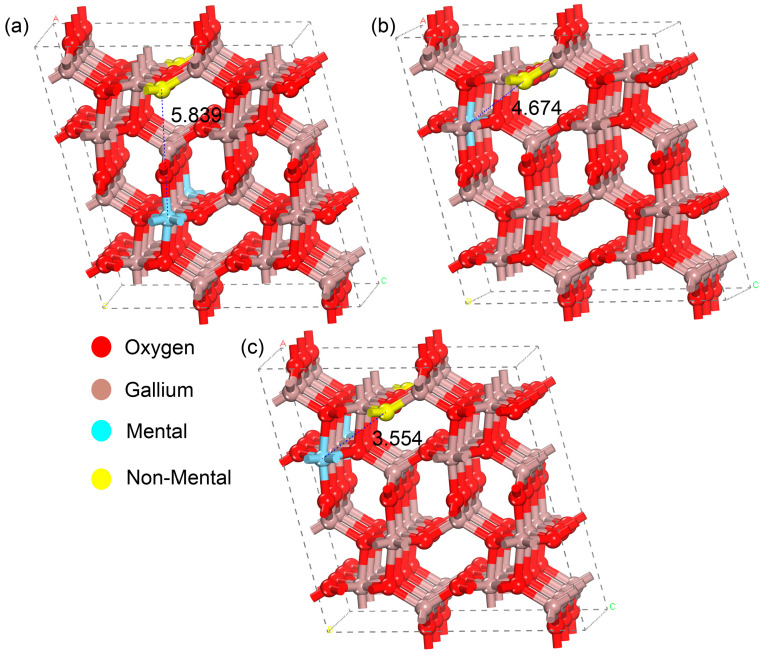
Schematic diagram of doping sites in the 1 × 3 × 2 supercell. (**a**) Doping site 1 (**b**) Doping site 2 (**c**) Doping site 3.

To investigate the stability of different doping sites, Table 1 lists the calculated lattice parameters, volume, and total energy ($E_{tot}$) of all doped systems. The increase in lattice volume is primarily attributed to the larger atomic radii of the selected Ag and S atoms compared to Ga and O atoms. The study found that among the (metal, non-metal) co-doped β-Ga_2_O_3_ systems, Ag_Ga2_-S_O3_ and Ag_Ga2_-Cl_O3_ exhibit lower total energies and greater stability compared to other systems. This indicates that metal atoms tend to occupy the Ga2 site, while non-metal atoms (Cl and S) prefer the O3 site. To further confirm the doping sites, as shown in Figure 2, we calculated the total energies for three different O3 positions. The distances between the Ag and Cl atoms in these configurations are 5.84 Å, 4.67 Å, and 3.55 Å, respectively, with corresponding total energies of −346.3258 eV, −345.9495 eV, and −346.3329 eV. The structural stability follows the order III > I > II. Therefore, the subsequent doping sites in this study are all based on configuration III.

To investigate the modulation mechanisms of Mg and Zn co-doped with S and Cl on the electronic structure and optical properties of β-Ga_2_O_3_, the first step is to evaluate the structural stability of the doped systems [39,40,41]. Research has shown that the thermodynamic stability of doped systems is primarily determined by the chemical environment around the atoms [42,43]. This property can be quantitatively characterized by the doping formation energy $E_{fcommon}[X^{q}]$ [44,45]. Based on this, the doping formation energy in this study is calculated using the following formula:

(1) $$E_{f\mathrm{common}}[X^{q}]=E_{tot}[X^{q}]-E_{tot}[bulk]+\mu_{\mathrm{Ga}}+\mu_{O}-\mu_{N}-\mu_{X}$$

$E_{tot}[X^{q}]$ and $E_{tot}[bulk]$ represent the total energies of the system with and without dopants, respectively. $\mu_{Ga}$ and $\mu_{O}$ denote the chemical potentials of Ga and O atoms, while $\mu_{X}$ and $\mu_{N}$ represent the chemical potentials of the metal and non-metal dopants. Considering the differences in formation energy under oxygen-rich and gallium-rich conditions, we calculated the formation energies for both scenarios separately. The chemical potentials of O and Ga atoms under different conditions satisfy the following formulas:

(2) $$2\mu_{Ga}+3\mu_{O}=\mu_{Ga_{2}O_{3}}$$

(3) $$\Delta \mu_{Ga}=\mu_{Ga}-E_{Ga}$$

(4) $$\Delta \mu_{O}=\mu_{O}-\frac{1}{2}E_{O_{2}}$$

**Figure 3:**
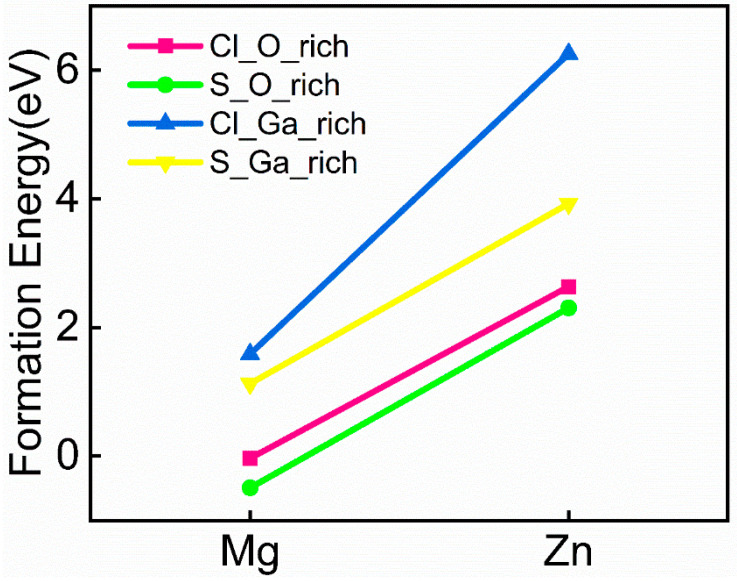
Formation energies of co-doped systems.

After determining the doping sites, we calculated the formation energies under both oxygen-rich and gallium-rich conditions. Figure 3 shows the formation energies of (metal, non-metal) co-doped systems under different chemical environments. By comparing the results, we found that the co-doped structures exhibit lower formation energies and greater stability under oxygen-rich conditions, making them easier to form.

### 3.2. Electronic Properties

**Figure 4:**
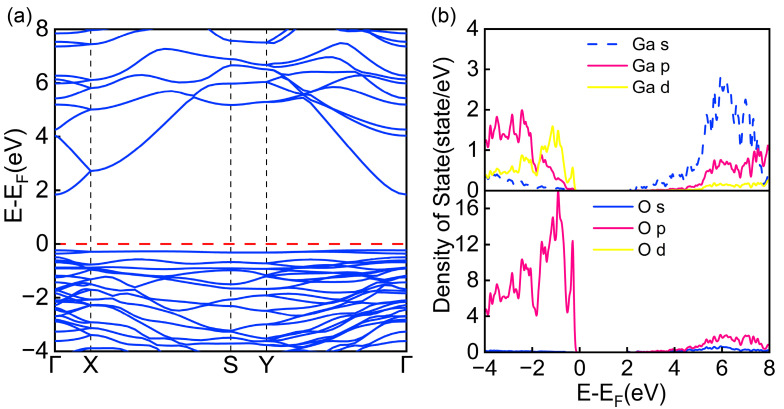
(**a**) Band structure of β-Ga_2_O_3_. (**b**) Partial density of states (PDOS) of β-Ga_2_O_3_.

**Figure 5:**
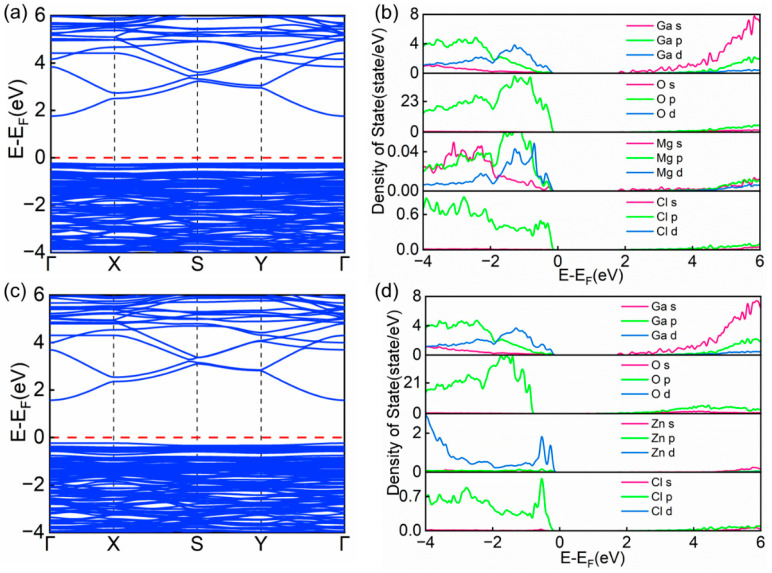
(**a**) Band structure of Mg_Ga2_-Cl_O3_. (**b**) Partial density of states (PDOS) of Mg_Ga2_-Cl_O3_. (**c**) Band structure of Zn_Ga2_-Cl_O3_. (**d**) Partial density of states (PDOS) of Zn_Ga2_-Cl_O3_.

**Figure 6:**
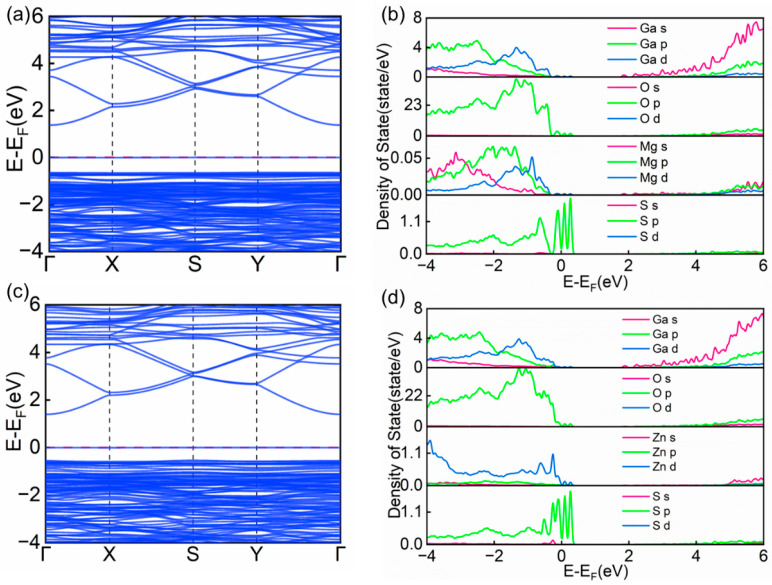
(**a**) Band structure of Mg_Ga2_-S_O3_. (**b**) Partial density of states (PDOS) of Mg_Ga2_-S_O3_. (**c**) Band structure of Zn_Ga2_-S_O3_. (**d**) Partial density of states (PDOS) of Zn_Ga2_-S_O3_.

The physical properties of solid materials can typically be characterized by analyzing their band structures and partial density of states (PDOS) [46,47]. To gain a deeper understanding of the influence of Mg and Zn co-doped with Cl and S on the electronic properties of β-Ga_2_O_3_, this study systematically examined the band structure features and PDOS distributions of the doped systems. As shown in Figure 4a, Figure 5a,c and Figure 6a,c, the band structure characteristics of intrinsic β-Ga_2_O_3_ were compared with those of (Mg, Cl), (Mg, S), (Zn, Cl), and (Zn, S) co-doped systems, the red dashed part of the figure shows the calibrated Fermi energy levels. The analysis revealed that the calculated bandgap of intrinsic β-Ga_2_O_3_ is 2.08 eV (Figure 4a), which is slightly lower than experimental measurements [48] but consistent with existing theoretical results [49]. Along the Brillouin zone path Γ → X → S → Y → Γ, both the valence band maximum and conduction band minimum of intrinsic β-Ga_2_O_3_ are located at the Γ point, confirming that β-Ga_2_O_3_ is a direct bandgap semiconductor.

When metal elements (Mg, Zn) and non-metal elements (Cl, S) are co-doped into the β-Ga_2_O_3_ semiconductor lattice, significant changes occur in the band structure, as shown in the results. As shown in Table 2, the calculated bandgap values for the Mg_Ga2_-Cl_O3_ and Zn_Ga2_-Cl_O3_ systems are 1.99 eV and 1.81 eV, respectively. In comparison, the bandgap values for the Mg_Ga2_-S_O3_ and Zn_Ga2_-S_O3_ systems are 2.02 eV and 1.95 eV, respectively. Notably, the bandgap values of these co-doped systems are all lower than that of undoped β-Ga_2_O_3_. This phenomenon indicates that the synergistic doping of metal elements with non-metal elements (Cl, S) can effectively modulate the bandgap of β-Ga_2_O_3_, leading to a significant reduction in its bandgap value.

Experimental results show that the doping of Mg, Zn, Cl, and S atoms causes a noticeable low-energy shift in the conduction band of β-Ga_2_O_3_. This co-doping effect significantly enhances the probability of electron transitions between the conduction band minimum and valence band maximum near the Fermi level, providing a reasonable explanation for the observed bandgap reduction. Notably, all four doping configurations introduce new impurity levels near the top of the valence band.

To gain a deeper understanding of the electronic properties of the doped systems, Figure 4b, Figure 5b,d and Figure 6b,d systematically present the partial density of states (PDOS) distributions of intrinsic β-Ga_2_O_3_ and its co-doped systems. Analysis reveals that in intrinsic β-Ga_2_O_3_, the O-2p orbitals primarily contribute to the formation of the valence band, while the Ga-4s orbitals dominate the conduction band. This characteristic indicates significant s-p orbital hybridization near the Fermi level. It is precisely this electronic interaction between Ga and O atoms that forms stable Ga-O chemical bonds, thereby enhancing the structural stability of the β-Ga_2_O_3_ crystal.

From the partial density of states (PDOS) analysis in Figure 5b,d and Figure 6b,d, it can be seen that the contributions of Ga and O atoms to the valence and conduction bands in the co-doped systems do not significantly differ from those in intrinsic β-Ga_2_O_3_. However, the introduction of dopants causes varying degrees of low-energy shifts in the orbital densities of states for both Ga and O atoms. Specifically, within the valence band range of −4 eV to 0 eV, the Mg-s, Mg-p, Mg-d, and Zn-d orbitals introduce new energy level features in their respective co-doped structures. Additionally, the p orbitals of the non-metal elements S and Cl contribute extra impurity levels within this energy range. These newly introduced energy level features have a significant impact on the electronic structure of the system.

### 3.3. Effective Mass Analysis

The transport properties of semiconductor materials are primarily determined by the mobility of charge carriers. In theoretical studies, carrier mobility is often difficult to measure directly and is typically estimated indirectly using related parameters such as effective mass. Existing research has confirmed an inverse relationship between carrier mobility and effective mass. Based on the findings in reference [50], the effective mass can be calculated using the following formula:

(5) $$m*={ћ}^{2}{\left[\frac{\partial^{2}\varepsilon \left(k\right)}{\partial k^{2}}\right]}^{-1}$$

**Figure 7:**
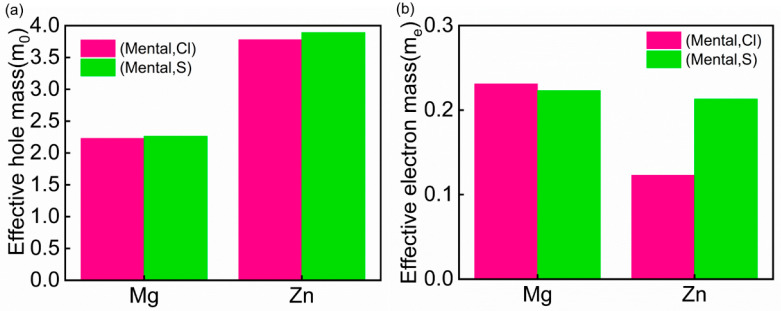
(**a**) Effective mass of holes. (**b**) Effective mass of electrons.

Figure 7 presents the calculated effective masses of charge carriers in the (metal, non-metal) co-doped β-Ga_2_O_3_ systems. The effective mass values in this study were calculated along the [100] crystallographic direction (from the Z point to the Γ point). The results show that the hole effective mass of undoped β-Ga_2_O_3_ is approximately 4.5 m_0_, while the electron effective mass is 0.45 m_0_, consistent with theoretical results reported in references [51,52]. Notably, the introduction of co-dopants significantly alters the effective masses of charge carriers, leading to a noticeable reduction in the hole effective mass.

### 3.4. Optical Properties

To investigate the effects of (metal, non-metal) co-doping on the optical properties of β-Ga_2_O_3_, this study examines the absorption coefficient and dielectric function [53,54]. The optical parameters of semiconductors are related to the complex dielectric function, which is expressed as follows [55,56]:

(6) $$E(\omega )=E_{1}(\omega )+iE_{2}(\omega )$$

(7) $$\varepsilon_{1}(\omega )=1+\frac{2}{\pi }\rho_{0}\int_{0}^{\infty }\frac{\omega^{\prime }\varepsilon_{2}(\omega )}{{\omega^{\prime }}^{2}-\omega^{2}}d\omega$$

(8) $$\varepsilon_{2}(\omega )=\frac{c}{\omega^{2}}\sum_{V,C}\int_{BZ}\frac{2}{{\left(2\pi \right)}^{2}}{\left|M_{C,V}(\kappa )\right|}^{2}\cdot \delta (E_{C}^{K}-E_{V}^{K}-\omega ћ)d^{3}\kappa$$

(9) $$\alpha (\omega )=\sqrt{2}\omega {\left[\sqrt{\varepsilon_{1}^{2}(\omega )+\varepsilon_{2}^{2}(\omega )}-\varepsilon_{1}(\omega )\right]}^{\frac{1}{2}}$$

$\varepsilon_{1}(\omega )$ and $\varepsilon_{2}(\omega )$ represent the real and imaginary parts, respectively. The real part is associated with the degree of electron polarization and is determined using the Kramer–Kronig relation. All other optical properties can be derived from $\varepsilon_{1}(\omega )$ and $\varepsilon_{2}(\omega )$ through the Kramer–Kronig relation. The real part of the dielectric function, $\varepsilon_{1}(\omega )$, can be obtained from Equation (7), the imaginary part, $\varepsilon_{2}(\omega )$, from Equation (8) [57], and the absorption coefficient from Equation (9) [58].

**Figure 8:**
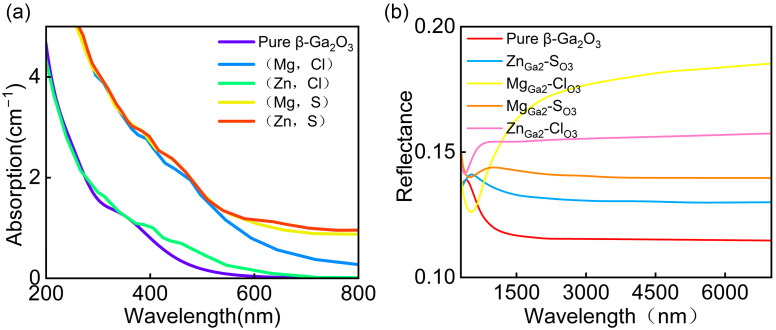
(**a**) Optical absorption coefficients of the systems. (**b**) Reflectance spectrum.

Figure 8a presents the optical absorption spectra and dielectric functions of intrinsic β-Ga_2_O_3_ and the co-doped systems (Mg_Ga2_-Cl_O3_, Zn_Ga2_-Cl_O3_, Mg_Ga2_-S_O3_, and Zn_Ga2_-S_O3_). Due to the underestimation of the bandgap by the GGA functional, the calculated spectra exhibit a significant red shift. However, the results show that the optical absorption intensity of the co-doped systems in the range of 200–800 nm is enhanced compared to that of intrinsic β-Ga_2_O_3_. Specifically, the absorption coefficients of Zn_Ga2_-S_O3_, Mg_Ga2_-S_O3_, and Mg_Ga2_-Cl_O3_ are significantly higher than that of intrinsic β-Ga_2_O_3_. This indicates that co-doping can effectively improve the visible light absorption capability and optical performance of β-Ga_2_O_3_ semiconductors.

Figure 8b compares the reflectivity of undoped β-Ga_2_O_3_ with that of Mg_Ga2_-Cl_O3_, Zn_Ga2_-Cl_O3_, Mg_Ga2_-S_O3_, and Zn_Ga2_-S_O3_ co-doped systems. The analysis reveals that in the mid-infrared range (3000~7000 nm), Mg_Ga2_-Cl_O3_-doped β-Ga_2_O_3_ exhibits the highest reflectivity, which is more than twice that of intrinsic β-Ga_2_O_3_. Additionally, the reflectivity of Zn_Ga2_-Cl_O3_, Mg_Ga2_-S_O3_, and Zn_Ga2_-S_O3_ co-doped β-Ga_2_O_3_ is significantly higher than that of the undoped system. These results suggest that co-doped β-Ga_2_O_3_ holds potential for applications in mid-infrared optical devices.

## 4. Conclusions

In this study, we systematically investigated the effects of Mg-Cl, Mg-S, Zn-Cl, and Zn-S co-doping on the structural stability, electronic, and optical properties of β-Ga_2_O_3_ semiconductor materials using first-principles calculations. Based on structural symmetry, we constructed and analyzed six different doping models. The main conclusions are as follows:

(1) The results show that the Mg-Cl, Mg-S, Zn-Cl, and Zn-S co-doping systems exhibit good thermodynamic stability in β-Ga_2_O_3_, particularly under oxygen-rich conditions, where the stability is more pronounced.

(2) Co-doping significantly reduces the bandgap of β-Ga_2_O_3_. Specifically, the bandgap of the Mg_Ga2_-Cl_O3_ co-doped system decreases by 4% (1.99 eV), the Zn_Ga2_-Cl_O3_ co-doped system by 12% (1.81 eV), the Mg_Ga2_-S_O3_ co-doped system by 3% (2.02 eV), and the Zn_Ga2_-S_O3_ co-doped system by 6% (1.95 eV).

(3) The Mg-Cl, Mg-S, Zn-Cl, and Zn-S co-doped β-Ga_2_O_3_ systems demonstrate enhanced optical absorption in the visible light region and significantly improved electrical conductivity.

Based on these findings, we believe that Mg-Cl, Mg-S, Zn-Cl, and Zn-S co-doped β-Ga_2_O_3_ have great potential for applications in solar cell photoelectrode materials, conductive thin films, high-absorption materials, electronic integrated circuit devices, and thermal infrared materials.

## Figures and Tables

**Table 1:** Lattice parameters, angles, volumes, and energies of the systems.

Structure	a (Å)	b (Å)	c (Å)	β°	V (Å^3^)	*E_tot_*
β-Ga_2_O_3_ (This work)	12.39	3.07	5.86	103.72	217.99	
Expt.1 [29]	12.55	3.08	5.89	103.67	222.43	
Expt.2 [30]	12.27	3.05	5.82	103.82	212.14	
Ag_Ga1_-S_O1_	13.27	3.10	5.89	105.12	234.68	−108.58
Ag_Ga1_-S_O2_	12.95	3.10	6.05	104.23	235.87	−108.92
Ag_Ga1_-S_O3_	12.56	3.09	6.09	102.24	231.41	−108.89
Ag_Ga2_-S_O1_	12.62	3.16	5.99	102.89	234.02	−109.35
Ag_Ga2_-S_O2_	12.83	3.15	6.06	103.72	240.53	−108.89
Ag_Ga2_-S_O3_	12.72	3.12	6.05	102.77	235.73	−110.12
Ag_Ga2_-Cl_O1_	12.61	3.14	6.12	103.32	237.34	−108.81
Ag_Ga2_-Cl_O2_	12.95	3.15	6.02	102.83	240.62	−108.06
Ag_Ga2_-Cl_O3_	12.67	3.15	6.07	102.86	237.68	−109.42

**Table 2:** Band structure of β-Ga_2_O_3_ before and after co-doping.

Model	CBM (eV)	VBM (eV)	Eg (eV)
Pure	1.84	−0.23	2.08
Mg-Cl	1.75	−0.23	1.99
Mg-S	1.37	−0.64	2.02
Zn-Cl	1.57	−0.23	1.81
Zn-S	1.39	−0.55	1.95

## Data Availability

The data can be shared and applied, and the corresponding author can be approached to obtain the data if there is a legitimate need for it by the researcher concerned.
